# Androgen levels in the fetal cord blood of children born to women with polycystic ovary syndrome: a meta-analysis

**DOI:** 10.1186/s12958-020-00634-8

**Published:** 2020-08-11

**Authors:** Changling Duan, Tianjiao Pei, Yujing Li, Qi Cao, Hanxiao Chen, Jing Fu

**Affiliations:** 1 Department of Obstetrics and Gynecology, West China Second University Hospital of Sichuan University, Chengdu, Sichuan 610041 PR China; 2 Key Laboratory of Birth Defects and Related Diseases of Women and Children (Sichuan University), Ministry of Education, West China Second University Hospital of Sichuan University, Chengdu, Sichuan 610041 PR China

**Keywords:** Polycystic ovary syndrome, Androgen, Cord blood, Infant, Meta-analysis

## Abstract

**Introduction:**

Polycystic ovary syndrome (PCOS) is one of the most common endocrine disorders in reproductive-aged women. It is reported that intrauterine exposure to hyperandrogenism may induce the development of PCOS and associated complications in later life. To analyze the intrauterine androgen levels in infants born to PCOS mothers, we evaluated the androgen levels in fetal cord blood through a meta-analysis of observational studies.

**Material and methods:**

The following online databases were systematically searched: PubMed, EMBASE, Cochrane library databases and Web of Science up to December 2019. Human studies compared cord blood androgen levels, including testosterone (T) and androstenedione (ADION), in fetal cord blood of mothers with and without PCOS. Statistical analysis was performed in Review Manager, Version 5.3, with the inverse variance method based on a random-effects model.

**Results:**

A total of 7 articles were scrutinized and a total of 570 samples including 268 female and 222 male infants were qualified for review. In the mass spectrograph (MS) subgroup, PCOS mothers showed no signs of increased T concentration in umbilical cord blood at birth (4 studies; hazard ratio [HR] = − 0.05; 95% confidence interval [CI] = [− 0.33,0.24]; I^2^ = 7%; *P* = 0.75; fixed-effects model). ADION level tends to be lower in daughters’ cord blood of PCOS mothers (3 studies; HR = -0.59; 95%CI = [− 1.00, − 0.19]; I^2^ = 0%; *P* = 0.004; fixed-effects model).

**Conclusions:**

Fetal cord blood T level is not related to PCOS, while ADION levels tend to be lower in the cord blood of daughters born to mothers with PCOS.

## Introduction

Polycystic ovary syndrome (PCOS) is one of the most common endocrine disorders, affecting up to 15% of reproductive-aged women [1]. PCOS is characterized by at least two of the following criteria: ovulatory dysfunction, polycystic ovary morphology, and signs of hyperandrogenism [2]. Besides, the syndrome leads to increased risks of metabolic disorders like insulin resistance and dyslipidemia, which in turn may cause the development of type 2 diabetes mellitus and cardiovascular disease [3].

However, the etiology of PCOS is still poorly understood. Franks et al. formulated a hypothesis that PCOS is a genetically determined ovarian pathology and is characterized by androgen overproduction. The heterogeneous manifestations present according to the interaction of the genetic “predisposition” with other genetic and environmental factors [4]. Recently, genetic and twin studies have demonstrated a high heritability of PCOS, particularly of hyperandrogenism [5–7]. Animal studies suggest that exposure to supraphysiological androgen concentrations in mothers may result in PCOS-like phenotypes in the offspring [8–10]. These studies strongly support Franks’ hypothesis that intrauterine exposure to hyperandrogenism may induce the development of PCOS and associated complications in later life. However, similar data in humans are insufficient.

It is reported that pregnant women with PCOS have increased serum androgen levels, including androstenedione and testosterone [11–14], which provides a potential source of fetal androgen excess. Insulin resistance in pregnant women with PCOS can lead to hyperinsulinemia, which may contribute to fetal hyperandrogenism exposure by inhibiting placental aromatase activity [13].

Umbilical cord blood endocrine characteristics reflect maternal, placental, and fetal endocrine conditions. Due to this, they have been used to determine the intrauterine fetal environment in recent studies on PCOS [11–13, 15–18]. It is almost certain that dehydroepiandrosterone (DHEA) in the fetal cord blood is not related to PCOS. However, the outcomes regarding testosterone (T) and androstenedione (ADION) are conflicting. Some studies have shown elevated T and/or ADION levels in the cord blood in the offspring of mothers with PCOS [15, 16, 18], but others have observed decreased ADION levels [13, 17] or no differences [11, 12] compared with controls.

Therefore, the aim of this meta-analysis was to determine the intrauterine fetal endocrine environment of PCOS by evaluating the androgen levels in fetal cord blood.

## Materials and methods

### Search strategy

A systematic literature search was conducted by two of the authors (Qi and Hanxiao). The PubMed, EMBASE, and Cochrane library databases as well as the Web of Science were searched using the following terms: “Polycystic Ovary Syndrome” and (“infant” or “pregnancy”) and “umbilical cord blood”. No language restrictions were applied. The last search was published in December 2019. Our study protocol has been submitted to PROSPETO (registration number: CRD42020125668).

### Inclusion criteria

Human studies that compared cord blood androgen levels, including those of T and ADION, in the fetal cord blood of mothers with and without PCOS were eligible for inclusion, provided that 1) participants in the PCOS group were diagnosed with PCOS based on the NIH or Rotterdam criteria, and the control group mothers did not have PCOS or PCOS-like symptoms.; 2) the study type could be cross-sectional, case-control or cohort studies; 3) the reports were full-text articles in English. For duplicate articles or data, the most comprehensive or the one published earliest were selected.

### Exclusion criteria

Studies were excluded when the titles or abstracts indicated that the papers were not relevant, or the papers were reviews, case reports, or other types without raw data. We conducted a quality assessment for the included studies; we used the Newcastle-Ottawa Scale (NOS) for case-control studies and the Agency for Healthcare Research and Quality (AHRQ) list for cross-sectional studies.

### Data extraction

Two of the authors (Tianjiao and Yujing) screened and evaluated all articles independently to extract the related information; differences of opinion were settled through negotiation. Furthermore, all the relevant references of the eligible original articles were examined to ensure data integrity. The collected data included the first names of the authors, year of publication, research type, diagnostic criteria, gestational age at delivery, primary study population, type of umbilical cord blood, method of assay, and the units of T and ADION. The scores of quality assessment were also recorded.

### Statistical analysis

Statistical analysis was performed using Review Manager, Version 5.3 (RevMan 5.3) with a two-sided *p*-value to compare cord blood androgen levels, including those of T and ADION, in the fetal cord blood of mothers with and without PCOS. For the included studies that used the Mann-Whitney U test, the median, the first and third quartiles were transformed to mean and standard deviation using the formula provided by Wan et al. [19]. Considering the variations in androgen assays and units among the studies, the standardized mean difference was selected to analyze the hazard ratios (HRs), 95% confidence interval (CI), and I-square (I^2^) using the inverse variance method. To prevent ignoring the possible heterogeneity between studies, we first used more conservative random-effects model rather than fixed effects. When I^2^ was not significant (< 50%), the fixed-effects model was applied. Moreover, a sensitivity analysis was conducted by deleting each study individually to evaluate the quality and consistency of the results. Subgroup analyses were performed according to assay types and the sex of the infants. Visual inspection of the funnel plot was done to assess possible publication bias.

## Results

### Included studies and characteristics

The search strategy identified 325 unique studies (among a total of 459) and 7 articles [11–13, 15–18] were scrutinized after literature retrieval and selection. A total of 570 samples including 268 female and 222 male (the rest 80 samples had no mention of sex) qualified for review.

**Fig. 1:**
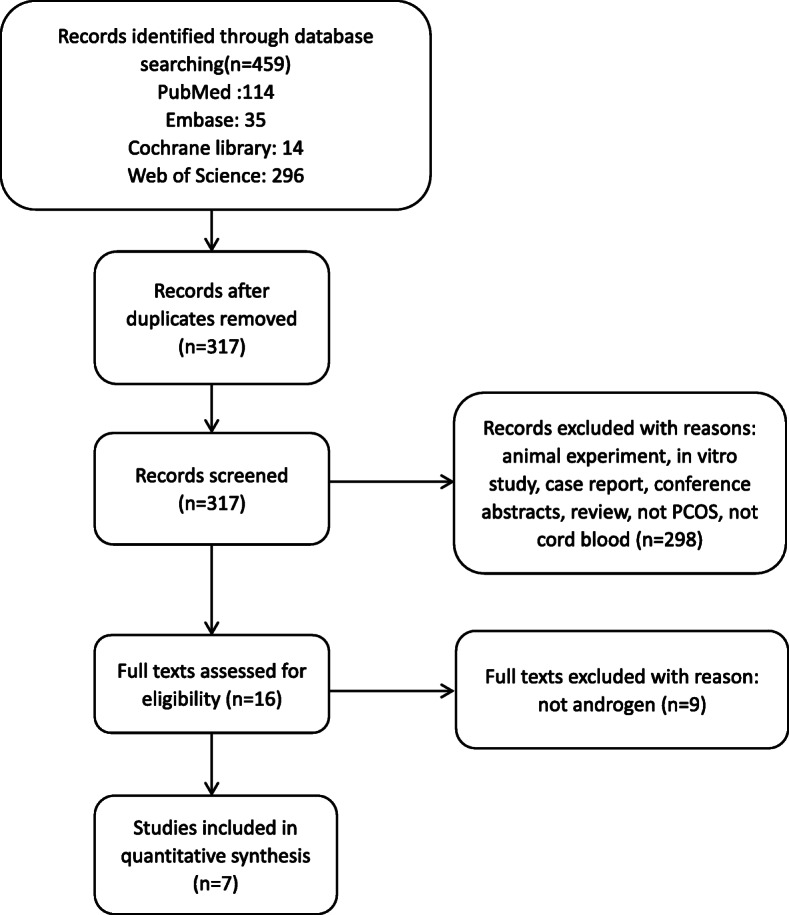
Flowchart of the study selection process

The included studies used various assays to measure serum androgen levels in the cord blood. Four studies [11, 12, 15, 17] used mass spectrograph (MS), others used multiple assays like chemiluminescent immunoassay (CLIA), direct electrochemiluminescence methodology (ECL), and radioimmunoassay (RIA). Two of the 7 studies [11, 16] were cross-sectional studies, the remaining 5 [12, 13, 15, 17, 18] were case-control studies. Most studies [12, 13, 15–17] collected mixed arteriovenous cord blood, Detti et al. obtained arterial and venous cord blood separately [11], while Barry et al. collected only venous cord blood [18]. The selection process is shown in Fig. 1, the characteristics and quality assessment scores of the eligible 7 studies are listed in Table 1.

**Table 1:** Characteristics and quality assessment scores of eligible studies

Author	Year	Research type	NOS or AHRQ score	Diagnostic criteria of PCOS	Gestational age	Primary study population	Type of cord blood	Testosterone		Androstenedione	
								Assay	Original unit	Assay	Original unit
Detti	2019	Cross-sectional	8	NIH criteria	term (≥38w)	PCOS vs non-PCOS women in labor	artery, vein	HPLC/MS	ng/dl	–	–
Daan	2017	Case-control	7	Rotterdam criteria	not mentioned	PCOS vs non-PCOS women outpatients	mixed	LC-MS/MS	nmol/L	LC-MS/MS	nmol/L
Caanen	2016	Case-control	8	Rotterdam criteria	not mentioned	PCOS vs non-PCOS in women a part of cohort study	mixed	LC-MS/MS	ng/ml	LC-MS/MS	ng/ml
Maliqueo	2013	Case-control	8	NIH and Rotterdam criteria	term (37-40w)	PCOS outpatients vs healthy women in 12th week of gestation	mixed	RIA	ng/ml	RIA	ng/ml
Mehrabian	2012	Cross-sectional	9	NIH criteria	term	PCOS vs healthy mothers who underwent elective cesarean section	mixed	CLIA	pg/ml	–	–
Barry	2010	Case-control	7	Rotterdam criteria	≥38w	PCOS vs non-PCOS women in labour wards	vein	ECL	nmol/L	–	–
Anderson	2010	Case-control	8	NIH criteria	>35w	PCOS vs non-PCOS women outpatients	mixed	LC-MS	ng/dl	LC-MS	ng/dl

*HPLC/MS* high-performance liquid chromatography/mass-spectrometry assay, *LC-MS/MS* liquid chromatography with tandem mass spectrometer, *RIA* radioimmunoassay, *CLIA* chemiluminescent immunoassay, *ECL* electrochemiluminescence methodology

### Association of PCOS and T in the fetal cord blood

**Fig. 2:**
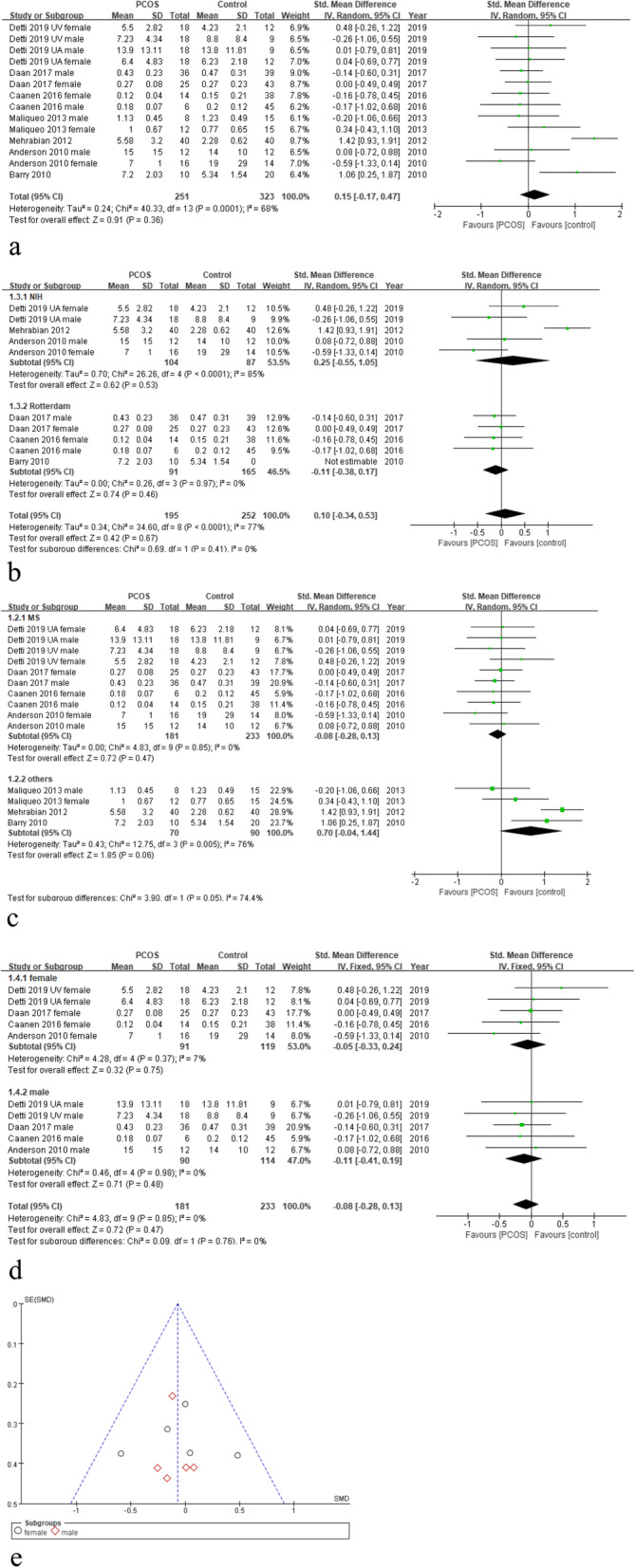
**a**-**c** Forest plot of testosterone levels difference in cord blood of PCOS and control groups: **a** all types of cord blood; **b** PCOS diagnosed by NIH and Rotterdam criteria; **c** measured using mass spectrograph (MS) and other assays; **d** measured using MS, collected from female and male infants. **e**: Funnel plot for the evaluation of potential publication bias related to testosterone levels measured using MS

Figure 2a shows the Forest plot of T levels difference in all types of cord blood of PCOS and control groups, including male and female newborns and mixed arteriovenous, arterial, and venous cord blood. The results demonstrate that there is no significant difference in cord blood T levels between two groups (7 studies; HR = 0.15; 95%CI = [− 0.17,0.47]; I^2^ = 68%; *P* = 0.36; random-effects model).

The differences of PCOS diagnosis criteria and assays of T between studies may have resulted in high heterogeneity (I^2^ = 68%). We conducted a subgroup analysis by PCOS diagnosis criteria, NIH and Rotterdam criteria. The results showed there is still a high heterogeneity in NIH subgroup (3 studies; HR = 0.25; 95% CI = [− 0.55,1.05]; I^2^ = 85%; *P* = 0.53; random-effects model; Fig. 2b). However, no heterogeneity was found in Rotterdam subgroup (3 studies; HR = -0.11; 95% CI = [− 0.38,0.17]; I^2^ = 0%; *P* = 0.46; random-effects model; Fig. 2b). In both subgroups, there is no significant difference of T level between PCOS and control cord blood (Fig. 2b).

As for subgroups divided by MS and other types of assays. The results showed no heterogeneity in the MS group (4 studies; HR = -0.08; 95% CI = [− 0.28,0.13]; I^2^ = 0%; *P* = 0.47; random-effects model; Fig. 2c). Then, we created a further grouping according to infants’ sex using the fixed-effects model, the result showed no significant difference in both subgroups (Fig. 2d). The funnel plot indicated that there is no visual publication bias (Fig. 2e).

### Association of PCOS and ADION in the fetal cord blood

**Fig. 3:**
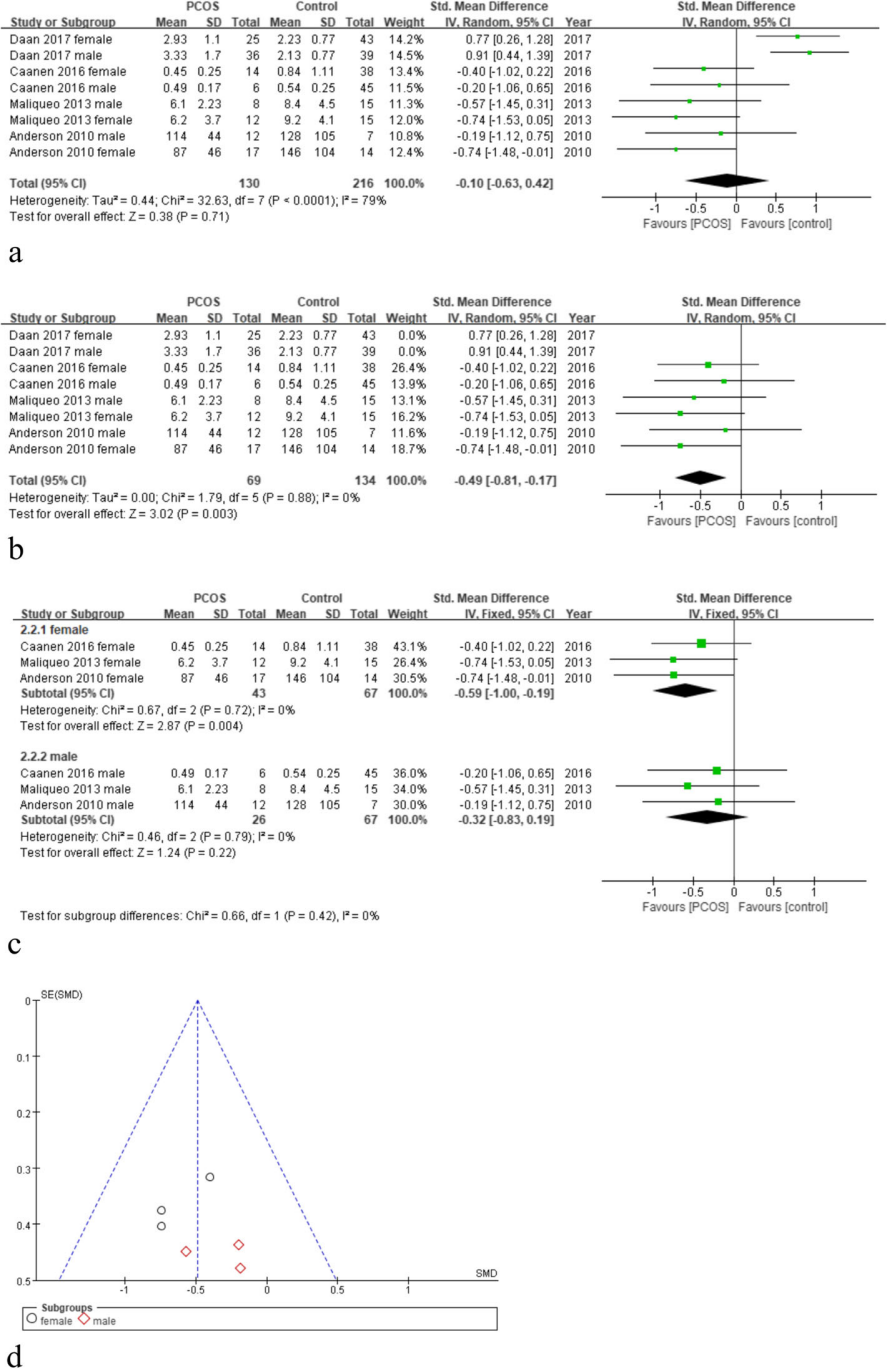
**a**-**c** Forest plot of androstenedione levels difference in cord blood of PCOS and control groups: **a** all eligible studies; **b** deleting Daan’s study; **c** collected from female and male infants after deleting Daan’s study. **d**: Funnel plot for the evaluation of potential publication bias related to androstenedione levels after deleting Daan’s study

The Forest plot of ADION levels difference in all types of cord blood is shown in Fig. 3a, the difference in cord blood ADION levels between PCOS and control mothers was not significant (4 studies; HR = -0.10; 95%CI = [− 0.63,0.42]; I^2^ = 79%; *P* = 0.71; random-effects model).

Sensitivity analysis was conducted by deleting each study individually. We found I^2^ = 0% after removing the study by Daan et al. (Fig. 3b). The subgroups were divided according to infants’ sex after deleting Daan’s study. The funnel plot also showed no bias (Fig. 3d). As presented in Fig. 3c, the ADION level in female newborns’ cord blood was significantly lower than that seen in the control group. In the male newborn group, the difference was not obvious.

## Discussion

In the present meta-analysis, we found that infants born to PCOS mothers showed no signs of increased T concentration in umbilical cord blood at birth, irrespective of their sex. The ADION level seemed to be decreased in the cord blood of daughters born to PCOS mothers. Regarding the T level, high heterogeneity was noted among all eligible 7 studies, which indicated a relationship with the PCOS diagnostic criteria and measurement mode. In studies eligible for ADION analysis, I^2^ was extremely high. After deleting Daan’s study, the I^2^ decreased to 0 and it is demonstrated that the ADION level was reduced in the cord blood of daughters born to PCOS mothers.

The diagnostic criteria of PCOS was first proposed in a 1990 meeting of the National Institutes of Health (NIH), which required both clinical/biochemical hyperandrogenism and chronic anovulation [20]. Based on NIH criteria, Rotterdam criteria added polycystic ovary (PCO) appearance on ultrasonography, at least two of the following three criteria were mandatory [2]. It is reported that the incidence of hyperandrogenism, IR and other endocrine metabolic disorders was different between PCOS patients diagnosed according to NIH standards and Rotterdam standards [21–23]. In our study, we found there is a high heterogeneity between studies using NIH criteria to diagnose PCOS, while no heterogeneity was found in Rotterdam subgroup. This indicated that Rotterdam criteria may be more appropriate of PCOS diagnose. Besides, in both subgroup analysis, there is no significant difference of T level between PCOS and control cord blood.

Recently, many laboratories have been using immunoassay-based methods, such as electrochemical techniques and chemiluminescence, to measure T. However, immunoassay has inevitable accuracy and precision problems [24, 25]. Actually, in the eligible studies using immunoassay, we found that the mean values of T level in fetal cord blood varies from 0.228 to 207.36 after converting all units to ng/dL [13, 16, 18]. It is reported that guessing testosterone concentrations are more accurate than some immunoassays for women [26]. Although the values of mass spectrometry have some variability, their precision and accuracy are far better than those of the immunoassays [27]. This may explain the low heterogeneity in the MS subgroup of T level’s analysis.

In our meta-analysis of ADION levels in the cord blood, significant heterogeneity was seen among the 4 eligible studies. The heterogeneity remained at a high level until we deleted Daan’s study, which decreased the I^2^ to 0. However, after carefully reading the full text of Daan’s study, no methodological reasons were found for the high heterogeneity. In the discussion part, Daan stated that the differences in their result when compared to those in other reports could potentially be influenced by variations in placental tissue activity among women with PCOS, along with differences in fetal adaptation [15].

Steroids cross the placenta in both directions and mostly are metabolized by aromatase. This reduces the passage of androgens between the maternal and fetal compartments. Our analysis suggests that fetal cord blood T level is not related to PCOS, which indicated that T of PCOS mothers may be quickly degraded and converted by the placenta’s aromatase into estradiol when passing through the placenta [11]. According to our results, ADION levels tend to be lower in the cord blood of daughters born to mothers with PCOS. Cord blood androgens are derived from both fetal adrenal as well as placental steroidogenesis. The fetal adrenals actively produce DHEAS in utero, which is transformed into ADION, testosterone, and estradiol by the placenta [17, 28]. It is possible that decreasing ADION level in the cord blood is related to the alterations of steroidogenesis in the fetus or an abnormality in placental steroidogenesis.

Furthermore, we found high heterogeneity and decreasing trend of ADION levels in daughters born to mothers with PCOS. In current clinical studies, the ADION level is not routinely assessed because of its inconclusive diagnostic value in PCOS [29]. However, a recent observational study has suggested that serum ADION level may be the most sensitive indicator of androgen excess in women with PCOS. Over two-thirds of PCOS women with normal testosterone levels were found to exhibit increased ADION levels in this study [30].

There are several limitations to our study. The limited number of studies is an inevitable factor affecting the reliability of the results. Especially regarding the ADION result, there are only 4 eligible studies with high heterogeneity. We have to carefully assess the reliability of the decreasing trend of ADION in the cord blood of daughters born to PCOS mothers. Besides, only 2 studies [11, 18] collected umbilical venous and arterial blood separately, so we did not conduct a subgroup of cord blood type for the blood type. Although there may be small variations between arterial and venous steroid concentrations, strong correlations have been previously described [31].

To our knowledge, this is the first meta-analysis on the relation between PCOS and androgen levels in fetal cord blood. Our study helps demonstrate the priority of MS in the measurement of umbilical cord blood testosterone. Prima facie, our analysis seems to fail to support the hypothesis that maternal androgen excess contributes to elevated T concentrations in the cord blood, and therefore the development of PCOS. However, it indicates the potential role of placenta aromatase in T transportation between fetus and mothers. Besides, umbilical cord blood can only represent T levels at the end of pregnancy rather than the entire gestation period. The decreasing ADION levels in cord blood also revealed the possibility of fetal adrenal and ovary steroidogenesis alterations. In the future, research on the effect of PCOS to their offspring is still important, the results of fetal androgen changes should be combined with the alterations of placental steroidogenesis and fetal steroidogenesis changes if possible.

## Conclusion

In conclusion, this meta-analysis suggests that fetal cord blood T level is not related to PCOS, while ADION tends to be reduced in cord blood of daughters born to mothers with PCOS. More studies on PCOS intrauterine ADION level changes and alterations of placental steroidogenesis are needed.

## Data Availability

The datasets used and analysed during the current study are available from the corresponding author on reasonable request.

## Abbreviations

ADION: Androstenedione
CI: Confidence interval
HRs: Hazard ratios
MS: Mass spectrograph
PCOS: Polycystic ovary syndrome
T: Testosterone
